# Complex spectrum of phenobarbital effects in a mouse model of neonatal hypoxia-induced seizures

**DOI:** 10.1038/s41598-018-28044-2

**Published:** 2018-07-03

**Authors:** Sean M. M. Quinlan, Natalia Rodriguez-Alvarez, Eleanor J. Molloy, Stephen F. Madden, Geraldine B. Boylan, David C. Henshall, Eva M. Jimenez-Mateos

**Affiliations:** 1 Department of Physiology & Medical Physics, Royal College of Surgeons in Ireland, 123, St Stephen Green, Dublin, 2 Dublin Ireland; 2 Paediatrics, Academic Centre, Tallaght Hospital, Trinity College, The University of Dublin, Dublin, Ireland; 3 Neonatology, Coombe Women and Infants’ University Hospital, Dublin, Ireland; 4 Neonatology, Our Lady’s Children’s Hospital Crumlin, Dublin, Ireland; 5 Data Science Centre, Beaux Lane House, Royal College of Surgeons in Ireland, Dublin, 2 Ireland; 6 Irish Centre for Fetal and Neonatal Translational Research (INFANT), Cork, Ireland; 7 Department of Paediatrics and Child Health, University College Cork, Cork, Ireland

## Abstract

Seizures in neonates, mainly caused by hypoxic-ischemic encephalopathy, are thought to be harmful to the brain. Phenobarbital remains the first line drug therapy for the treatment of suspected neonatal seizures but concerns remain with efficacy and safety. Here we explored the short- and long-term outcomes of phenobarbital treatment in a mouse model of hypoxia-induced neonatal seizures. Seizures were induced in P7 mice by exposure to 5% O_2_ for 15 minutes. Immediately after hypoxia, pups received a single dose of phenobarbital (25 mg.kg^−1^) or saline. We observed that after administration of phenobarbital seizure burden and number of seizures were reduced compared to the hypoxic period; however, PhB did not suppress acute histopathology. Behavioural analysis of mice at 5 weeks of age previously subjected to hypoxia-seizures revealed an increase in anxiety-like behaviour and impaired memory function compared to control littermates, and these effects were not normalized by phenobarbital. In a seizure susceptibility test, pups previously exposed to hypoxia, with or without phenobarbital, developed longer and more severe seizures in response to kainic acid injection compared to control mice. Unexpectedly, mice treated with phenobarbital developed less hippocampal damage after kainic acid than untreated counterparts. The present study suggests phenobarbital treatment in immature mice does not improve the long lasting functional deficits induces by hypoxia-induced seizures but, unexpectedly, may reduce neuronal death caused by exposure to a second seizure event in later life.

## Introduction

Seizures are most prevalent during the neonatal period, defined as the first 28 days of life in a term infant, affecting 3–5 in every 1000 live births^1^. The most common cause is hypoxic-ischemic encephalopathy and neonatal seizures are characterized by abnormal, repeated, paroxysmal alterations in neurological function (behavioural, motor and autonomic functions). The pathophysiology and semiology of seizures are different in the neonatal brain compared to the mature brain due to developmental factors such as incomplete myelination, immature synapse formation and neurochemical differences. Nevertheless, prolonged or repeated neonatal seizures are treated as a medical emergency because of concern they may be directly harmful to the developing brain^2,3^.

Diagnosis of seizures in the neonatal period remains a challenge, resulting in over-diagnosis and, as a consequence, over-treatment of infants^4^. Currently, phenobarbital (PhB) remains the first line drug therapy for the treatment of suspected neonatal seizures^5^. Indeed, the World Health Organization strongly recommended only the use of PhB as first-line treatment of neonatal seizures^6^, despite the low-quality evidence to support efficacy^7,8^. Concern around safety and efficacy of PhB is thought to relate to the mechanism of action, as PhB is a positive allosteric modulator of the γ-amino butyric acid (GABA)-A receptor. Activation of GABA-A receptor, in the neonatal brain, leads to efflux of Cl^−^ from immature neurons and results in depolarizing effects which may promote rather than oppose hyper-excitability^9^.

Over the last two decades, concerns have been raised about anti-epileptic drug (AED) administration in infants due to possible adverse effects on the immature brain^10–12^. Several studies have reported that treatment with PhB in children aged 8–36 months results in lower cognitive performance than un-treated children, without the benefit of seizure prevention^11,13^.

Studies in animal models also suggest that exposure to PhB during the neonatal period produces long-lasting structural and functional changes in the brain^14^. Indeed, acute exposure to PhB (20–40 mg.kg^−1^, as given in the clinical setting^12,15^), to post-natal day 7 (P7) rats resulted in apoptosis of neurons in the cortex and limbic system^12,16,17^. There is also evidence that PhB exposure in the neonatal period produces long-lasting behavioural changes in rodents. Studies have reported that immature rats given PhB displayed a range of anxiety-like and schizophrenic-like behaviours in adulthood, affecting all behavioural domains including cognitive, emotional and motor functions^14,18–20^.

In this study, for the first time, we sought to analyse the acute and chronic effects of PhB treatment in a mouse model of hypoxia-induced seizures^21^. In this model, P7 mice were exposed either to normoxia or hypoxia and PhB or vehicle (saline). To begin to elucidate the effects of PhB given after hypoxia-induced seizures, its acute effects as a treatment of neonatal seizures and subsequent neurological damage were analysed. Additionally, long-lasting effects of phenobarbital were assessed, behaviour (motor activity, anxiety-like behaviour and cognitive impairment) and seizure susceptibility. Our results corroborate other work indicating PhB exposure is probably harmful to the immature brain but also point, unexpectedly, to protection against later-life seizure-damage.

## Results

### Effects of phenobarbital on electrographic neonatal hypoxia-induced seizures

**Figure 1:**
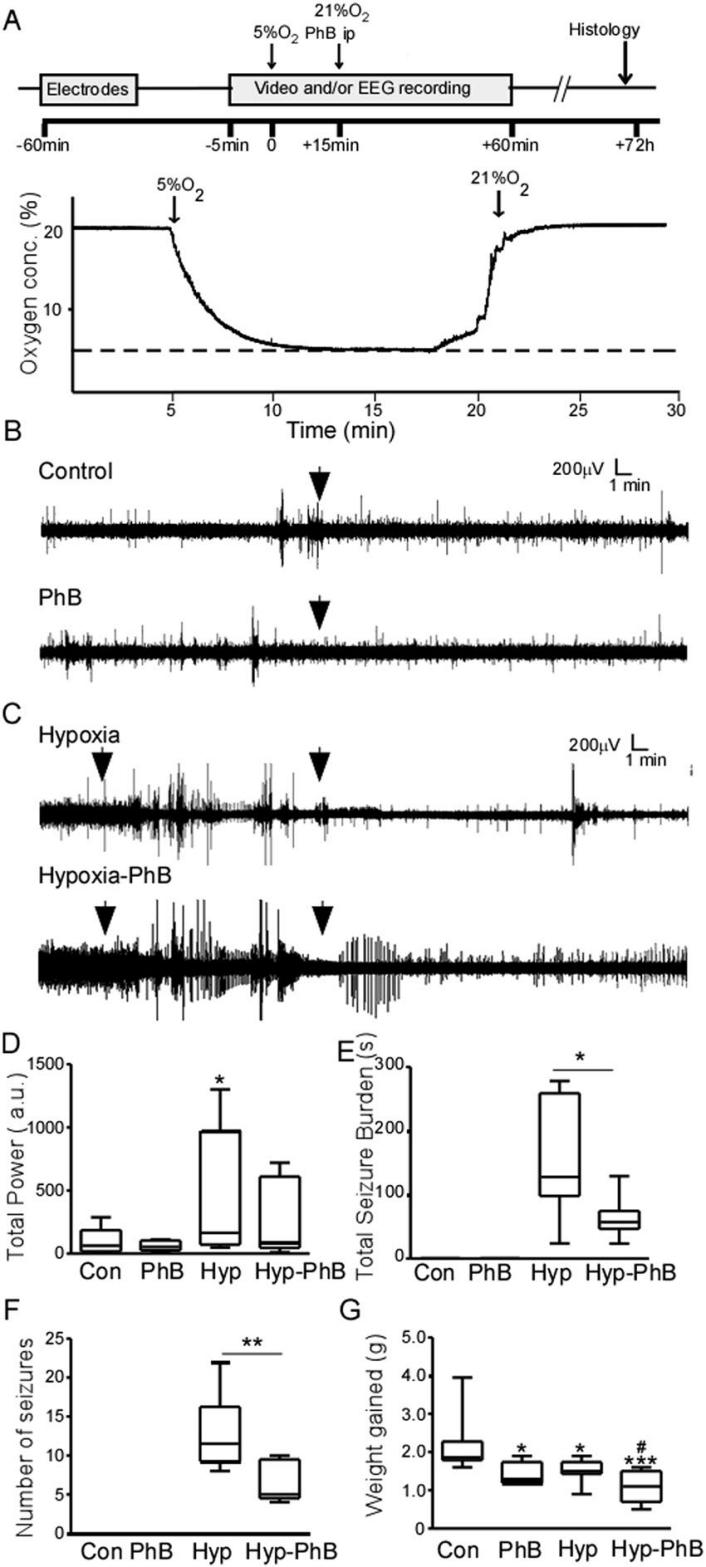
Effects of Phenobarbital on hypoxia induced neonatal seizures in P7 mice. (**A**) Experimental paradigm for induction of hypoxia (Hyp) and/or phenobarbital (PhB) treatment. Pups are placed in a warmed incubator (34 °C) and subject to 15 min of hypoxia. Immediately after, pups are injected with PhB 25 mg.kg^-1^ or saline vehicle. Pups under-going EEG recordings are sacrificed upon finishing and pups for histological analyses were returned to dams for 72 h. (**B**) Representative EEG traces from control receiving saline (top EEG) or PhB (bottom EEG) at P7. Note: Arrows indicate saline or PhB injection. (**C**) Representative EEG traces of hypoxia-exposed pups without (top EEG) or with PhB (bottom EEG). Note: the emergence of high-amplitude high-frequency discharges (HAHFDs) within minutes of hypoxia induction. Arrows indicate the period of hypoxia. (**D**) Quantification of EEG power compared to control pups. Evident that pups subject to hypoxia had a greater EEG power compared to control, phenobarbital and hypoxia-phenobarbital treated pups (n = 6–8 per group, ^*^P < 0.05 compared to control group). Hypoxia -phenobarbital pups had a greater EEG power compared to normoxic pups but not as great as untreated hypoxia exposed pups. (**E**,**F**) Hypoxia exposed mice, continued to experience seizure like activity with a higher number of seizures and an increase in seizure burden (n = 6–8 per group, ^*^P < 0.05 compared to control group), however normoxic mice did not. (**G**) Body weight gained 72 h after experiments at P7. Phenobarbital mice and hypoxia mice showed a similar reduced body weight gain after 72 h (n = 7 per group, ^*^P < 0.05, ^***^P < 0.001, ^#^P < 0.05, ^*^compared to control group, # compared to PhB or hypoxia group).

We first evaluated the effects of PhB on hypoxia-induced neonatal seizures in P7 mice, an age-appropriate model for neonatal seizures in humans^21–23^. P7 pups subjected to normoxia or hypoxia-induced seizures were injected with a single dose of vehicle or PhB (25 mg.kg^−1^, which falls within the normal dose used in the clinic (20–40 mg.kg^−1^)^15^ immediately upon re-oxygenation and electrographic activity was evaluated (Fig. 1A). In non-hypoxic animals, PhB had no obvious effects on EEG total power compared to control pups (Fig. 1B,D). Exposure of P7 pups to 15 min of global hypoxia resulted in electrographic and behavioural seizures, with these seizures continuing after pups were returned to room-air (Fig. 1B–F). In pups which received PhB after hypoxia-induced seizures, a significant reduction of EEG total power, total seizure burden and the number of seizures was observed (Fig. 1D–F). Since both responders and non-responders to PhB have been reported in previous studies we performed additional analysis to track individual responses to PhB within the group (Supp. Fig. 1A,B,C). This showed that hypoxia exposed pups, treated with vehicle, did not show a reduction in seizure burden post-hypoxia compared to the hypoxic period (Supp. Fig. 1A,C). In contrast, in the Hypoxia-PhB group 4 of the pups show a reduction in seizure burden after receiving PhB, compared to the hypoxia period (Supp. Fig. 1B,C).

### Body weight after phenobarbital and/or hypoxia-induced seizures

To further characterize the effects of PhB in the acute phase, body weight was assessed 72 h following exposure to the experimental paradigm. Control mice gained approximately 40% of their original body weight 72 h post-experiment (2 g) (Fig. 1G). In contrast, weight gain in mice that received PhB or were exposed to hypoxia was significantly lower (32.4% and 32.7% respectively (~1.5 g)) (Fig. 1G). Mice that were exposed to hypoxia and treated with PhB gained only 23% of their body weight (1 g), significantly less than all other groups, supporting an additive effect of hypoxia and PhB (Fig. 1G). To evaluate if the reduction of body weight gain is recovered later in life, mice were weighed again 5 weeks later. At 6 weeks old, no differences in weight gain were observed between groups (Con: 16.1 ± 0.8 g; PhB: 15.0 ± 0.9 g (p = 0.786 compared to control); Hyp: 13.9 ± 0.7 g (p = 0.3193 compared to control); Hyp-PhB: 15.4 ± 0.7 g (p = 0.9593 compared to control)).

### Phenobarbital-treated and hypoxia-exposed pups show neuronal damage in the hippocampus

**Figure 2:**
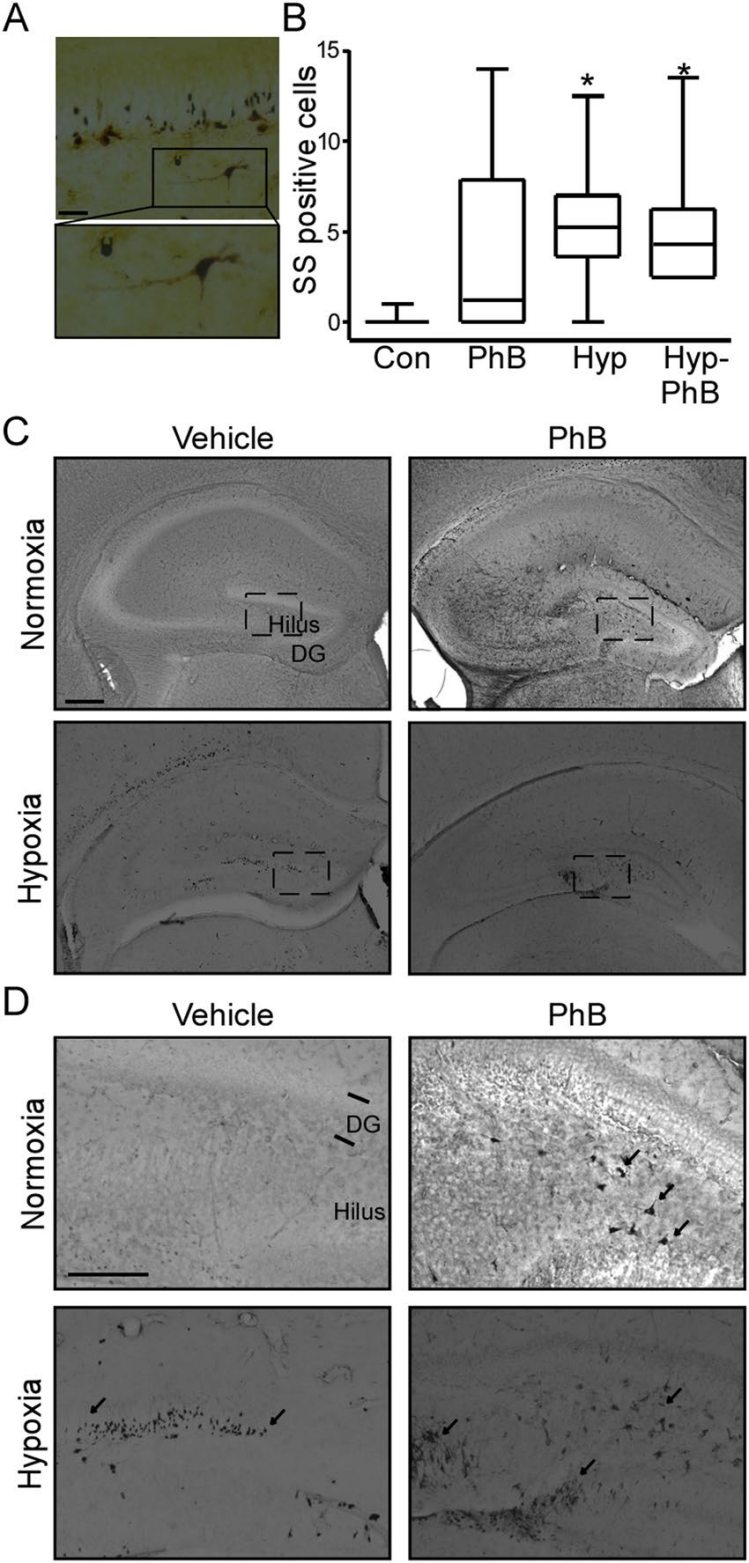
Neuronal damage after hypoxia-induced seizures in mice with/without phenobarbital (PhB) treatment at P7. (**A**) Representative high magnitude (40x) silver staining images from the hypoxia group. Scale bar: 50 μm. (**B**) Graph shows the number of silver-staining positive cells. Note: Both hypoxia-exposed groups have more silver-staining positive cells (n = 7, ^*^p < 0.05 compared to control). (**C**) Representative silver staining images of the hippocampus 72 h post-treatment. Note: Silver staining positive cells were mainly observed in the hilus of the phenobarbital, hypoxia and hypoxia-PhB treated groups. Scale bar: 200 μm. (**D**) Representative images of the hilus area of control, phenobarbital and/or hypoxia exposed mice. Scale bar: 200 μm.

Previously, assessment of neuronal damage using silver staining was observed as the most sensitive technique to evaluate neuronal damage at this developmental stage^21,24^ (Fig. 2A). We sought to analyse if PhB altered neuronal damage induced by hypoxia-induced seizures. Number of silver staining positive cells was very low in control pups, consistent with the limited physiological apoptosis at this age^12^ (Fig. 2B–D). PhB treated pups showed occasional silver-positive cells in the hilus although this did not differ statistically from counts in control pups. In contrast, silver positive cells were seen in both hypoxia-exposed groups, Hyp and Hyp-PhB mainly in the hilus (Fig. 2), with scattered positive cells in the CA1 and CA3 subfields (Fig. 2B,C). This finding suggests that PhB does not prevent the neuronal damage caused by hypoxia-induced seizures (Fig. 2B–D).

### Phenobarbital and/or hypoxia-induced seizures in neonates do not affect locomotor activity later in life

**Figure 3:**
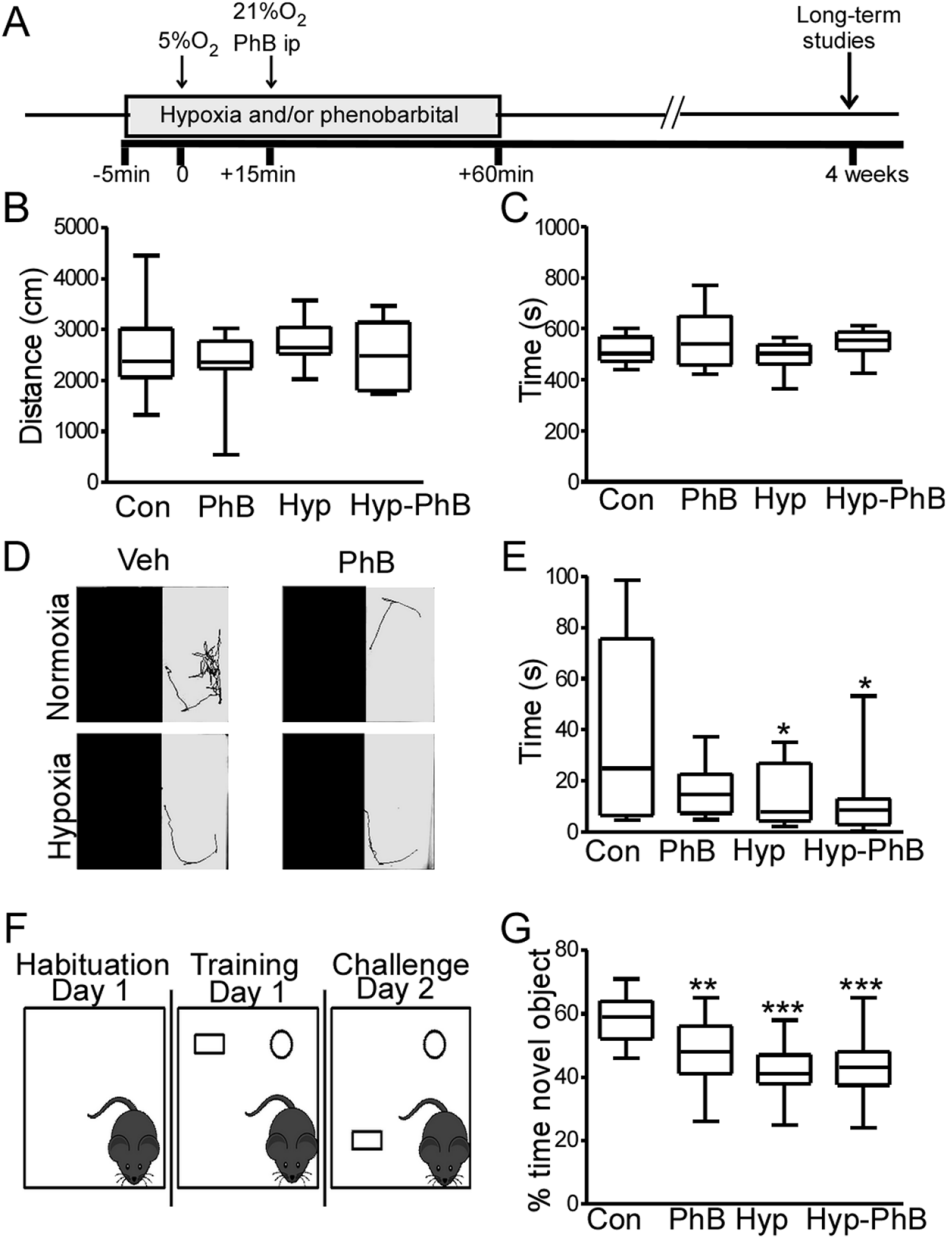
Locomotor, anxiety-like behaviour and hippocampal function in 5 weeks old mice previously subject to hypoxia and/or phenobarbital treatment at P7. (**A**) Four weeks after experiments at P7 mice were put through a battery of behaviour tests. (**B**,**C**) Locomotor activity was quantitatively assessed using the open-field task. There were no differences in parameters analysed; distance and time spent in the periphery (n = 10–12 per group, ^*^P < 0.05, ^**^P < 0.01, ^***^P < 0.001 compared to control group). (**D**,**E**) Anxiety-related behaviour was measured by latency of the mice to enter the dark area of the light-dark box task. (**D**) Representative images of mice tracking’s after being placed in the lit area of the dark box arena. Note: Control mice explored the brightly lit area more compared to other groups. (**E**) Confirmed by tracking software where treated mice moved directly into the dark area upon being placed in the arena (n = 10–12 per group, ^*^P < 0.05, ^**^P < 0.01, ^***^P < 0.001 compared to control group). (**F**) Schematic of the novel object-location task. After habituation to the arena mice are allowed to investigate 2 objects, after which, one of the objects is moved. (**G**) Analyses of the percentage of time mice spent interacting with the object showed that treatment groups spent less time with the novel located object compared to control mice (n = 10–12 per group, ^*^P < 0.05, ^**^P < 0.01, ^***^P < 0.001 compared to control group).

Separate groups of mice were next allowed to recover for at least 4 weeks and assessed at 5 weeks of age (Fig. 3A). First, the four different experimental groups (Con, PhB, Hyp and Hyp-PhB) were evaluated in an open arena (open-field task), and observed for 10 min.

The 10 min recording was binned into two 5 min recordings. Anxiety-like behaviours were analysed during the first 5 min of the task, while locomotor behaviours were analysed over the complete 10 min recording. There were no differences in parameters analysed (Fig. 3B,C and Supp. Fig. 2A–E). No differences were observed between groups in the distance moved (Fig. 3B, Supp. Fig. 2A), time spent in the periphery (Fig. 3C, Supp. Fig. 2B) or velocity of movement (Con: 3.9 ± 0.4 cm/s; PhB: 3.3 ± 0.3 cm/s; Hyp 4.1 ± 0.2 cm/s; Hyp-PhB: 3.6 ± 0.2 cm/s, Supp. Fig. 2C). Furthermore, no differences between groups were found in the freezing time (Con: 336 ± 25.3 s; PhB: 407.9 ± 35.7 s; Hyp: 322.3 ± 19.6 s; Hyp-PhB: 373.5 ± 20.1 s, Supp. Fig. 2D) and number of crossing between the centre and periphery area of the arena (Con: 60.5 ± 8.64; PhB: 48.5 ± 7.7; Hyp: 63.8 ± 6.0; Hyp-PhB: 48.7 ± 4.2, Supp. Fig. 2E).

### Phenobarbital treatment in neonates induces anxiety-like behaviour later in life and does not mitigate the long-lasting effect of hypoxia-induced seizures on anxiety-like behaviour

As a second assessment of anxiety-like behaviour, mice were tested in the light-dark box task, with latency to enter the dark area used as an index of anxiety-like behaviour^25^ (Fig. 3D,E). Control mice explored the light area for approximately 30–40 s before entering the dark area (Fig. 3D,E). In contrast, PhB and all hypoxia-exposed mice moved directly into the dark area following release in the light area (Fig. 3D,E). No differences in behaviour were found between the hypoxia and Hyp-PhB treated group (Fig. 3D,E).

### Deficits in hippocampal function after phenobarbital treatment at P7

We also assessed hippocampal function using the novel object-location task^26,27^ (Fig. 3F). Firstly, the total time spent with both objects was assessed. Control, PhB and hypoxia groups generally spent equal amounts of time interacting with both objects (control: 17.6 ± 1.9 s; PhB: 19.4 ± 1.8 s and Hyp: 19.4 ± 1.1 s). Hyp-PhB mice, however, spent less time with both objects compared to the other groups (14.3 ± 1.5 s, *p* = 0.03).

When the percentage of time with the novel object-location was evaluated, the control group spent 58% of the time with the novel object-location. In contrast, the three treated groups spent between 40–50% of the time interacting with the novel object (PhB: 47%; Hyp: 41% and Hyp-PhB: 44%) (Fig. 3F,G).

### Increased susceptibility to KA-induced seizures in mice treated with phenobarbital and/or hypoxia-induced seizures during the neonatal period

**Figure 4:**
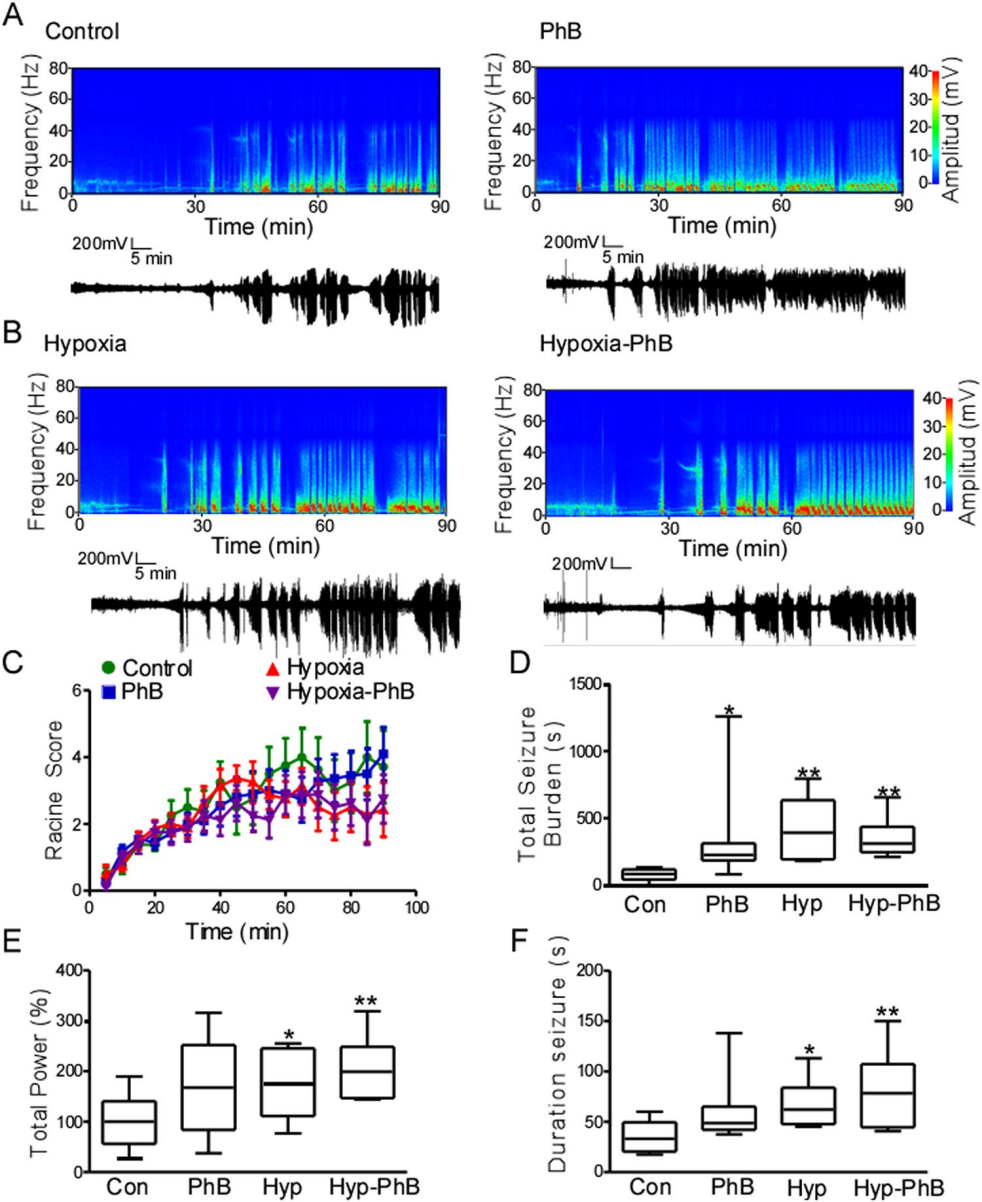
Reduced threshold to develop seizures later in life in mice after hypoxia-induced seizures and phenobarbital treatment in the neonatal period. 5 weeks after neonatal treatment, mice were implanted with electrodes and challenged with systemic KA (15 mg.kg^-1^), EEG and behaviour were recorded. (**A**,**B**) Representative spectrograms and corresponding EEG traces. (**C**) Seizure behavioural score taken every 5 min over the 90 min period using the Racine-like scale for mice. (**D**–**F**) Quantification of total seizure burden, total power and seizure duration during the 90 min of recording. (**D**) Seizure burden was higher in phenobarbital treated and hypoxia-induced mice compared to the control group (n = 8–11,^*^P < 0.05 ^**^P < 0.01 compared to control group). (**E**) Similar results was found in EEG power and (**F**) treated mice developed on average longer seizures (n = 8–11,^*^P < 0.05 ^**^P < 0.01 compared to control group).

To test whether responses to convulsive stimuli are altered in those mice exposed to phenobarbital and those that experienced neonatal seizures with or without PhB treatment, mice were injected with a low dose of KA (15 mg.kg^−1^ i.p.) at 6 weeks of age. EEG and behaviour were video-recorded for 90 min after KA injection (Fig. 4A,B). Upon KA injection, seizure behaviour was assessed using a modified Racine score^21^.

Similar to earlier reports^28^, no differences in behaviour were observed between groups during the 90 min recording (Fig. 4C). EEG analyses showed that mice developed typical ictal/epileptiform discharges 20–30 min post KA injection, independent of treatment (Fig. 4A,B). Significantly greater seizure burden and EEG power were observed in PhB, Hyp and Hyp-PhB mice compared to control mice (Fig. 4D,E). Although the seizure burden was similar across treated groups, when the duration of an individual seizure was evaluated, hypoxia exposed mice with or without PhB had longer seizures (Fig. 4F).

### Neuronal death following KA challenge is increased in mice previously subjected to hypoxia-induced seizures in the neonatal period

**Figure 5:**
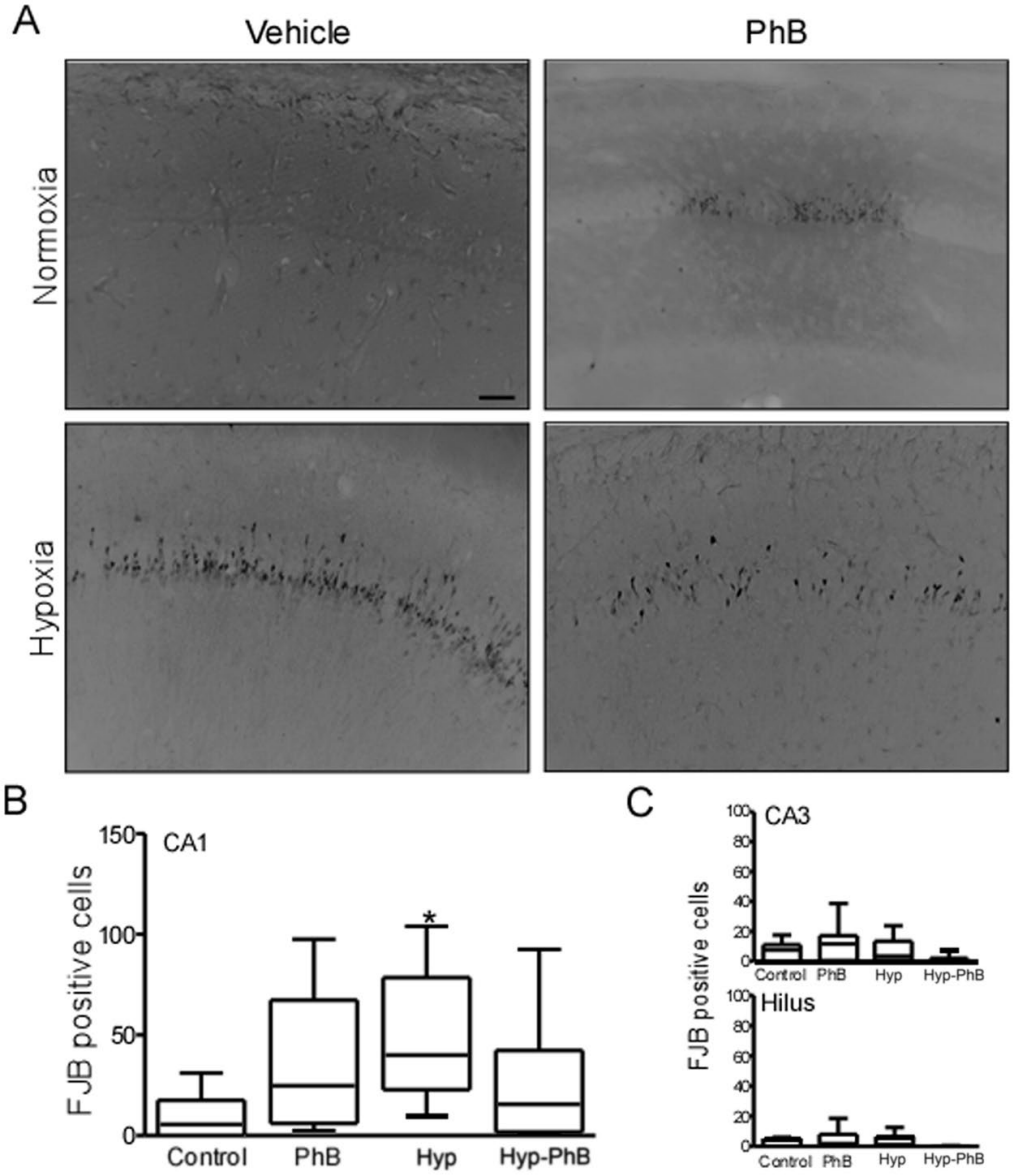
Assessment of neuronal injury 72 h post KA challenge. (**A**,**B**) Representative 20x photomicrographs of Fluoro-Jade B staining following KA challenge. Hippocampal sections show positive FJB positive cells mainly in the CA1 region of all groups, hypoxia mice show the greatest number of FJB positive cells. Scale bar: 100 μm. (**B**,**C**) Graph shows number of FJB positive cells in the CA1 subfield of the hippocampus (**B**), CA3 (**C**, Top) and Hilus (**C**, Bottom) (n = 6–7 per group ^*^p < 0.05 compared to control group). Note: Hypoxia exposed mice show an increase in FJB positive cells in the CA1 subfield of the hippocampus, no differences were observed in the CA3 and Hilus.

We have reported that, in contrast to control mice, hypoxia-exposed mice in the neonatal period show an increase of neuronal death after injection of intraperitoneal KA^21^. Now, we aimed to elucidate if PhB protects against this second hit. Brains were cut 72 h post-KA injection (when maximum neuronal death is observed), and FJB staining was carried out, as a marker of neuronal death in the mature brain. As expected, in the CA1 subfield of the hippocampus, hypoxic mice showed an increase in neuronal death (Fig. 5A,B). In contrast, no difference in neuronal death was observed in both phenobarbital-treated groups compared to the control group. Sporadic neuronal death was observed in the CA3 and Hilus in the four groups (Fig. 5C).

## Discussion

Phenobarbital continues to be the first line drug treatment of hypoxia-induced neonatal seizures despite on-going concerns with efficacy and safety in the developing brain. Using our animal model we have found that phenobarbital has a restricted response rate, while also causing adverse neurological outcomes later in life. We see neuronal damage 72 h after hypoxia-induced seizures and phenobarbital treatment; and long-lasting neuro-developmental effects, including anxiety-like behaviour and hippocampal-dependent memory. These findings suggest that a single clinically relevant dose of phenobarbital in the neonatal period does not normalize the acute or long lasting effects of hypoxia.

The poor response profile of phenobarbital in humans has been reported before, where approximately only 50% of patients will respond to PhB^8,29^. Similar to these observations, we saw a reduction in seizure activity in 50% of pups receiving 25 mg.kg^−1^ PhB. In the present study, we did not investigate potential sex differences in seizure susceptibility and response to PhB. Males have been reported to be more susceptible to seizures in some models^30^. Future studies will be necessary to investigate whether phenobarbital responses differ between male and female pups.

Despite the reduction in seizure activity, all pups receiving this dose or in combination with hypoxia displayed acute neuronal damage when analysed by silver staining. Although studies suggest it is possible to identify necrotic and apoptotic cells using silver staining, we did not undertake such analysis here. We observe that PhB did not ameliorate or aggravate the neuronal damage caused by hypoxia. Previously, PhB has been shown to induce neuronal damage 24 h after administration^12^. We need to consider that a peak of neuronal death induced by PhB may happen at an earlier time point. Finally, we did not explore why PhB induces potentially reversible neuronal damage. However, PhB reduces levels of the neurotrophic factors BDNF (brain-derived neurotrophic factor) and NT-3 (neurotrophin-3)^12^. During early brain development, both neurotrophic factors are necessary for survival of neurons, and, as a consequence, reduction of BDNF and NT-3 may have contributed to the increase in neuronal damage, as observed in PhB treated pups.

There is limited data available concerning the outcomes of PhB given in the clinic; however some studies have shown that children treated with PhB for febrile seizures display lower IQ scores, deficits in attention and reduced processing efficiency compared to untreated infants^31^. Consistent with these clinical observations, we have found that PhB exposure, in the neonatal period, results in a specific anxiety-like behaviour phenotype and impaired hippocampal function later in life. In the light-dark box task, control mice explored the arena before entering the dark area; however, Hyp and Hyp-PhB groups entered the dark area almost immediately, suggesting an anxiety-like behaviour. In the novel object-location test, the three treated groups showed altered hippocampal function compared to the control group. Both tests require intact hippocampi at function, as anxiety-like behaviours are controlled, among other brain regions, by the ventral hippocampus and the dorsal hippocampus is involved in memory^32^. Our results suggest that in the neonatal period, the hippocampus may be one of the affected brain regions following hypoxia-induced seizures^33^. Moreover these data indicate lasting effects of PhB administration exceeding the neuronal damage seen early after seizures. Further studies would be required to analyse whether the cell damage seen 72 h after hypoxia and/or PhB treatment could contribute to these behavioural phenotypes. Indeed, long-term behavioural effects of PhB have been reported in young rats^19^. Treatment of rats with PhB from P7 to P13 produced a similar increase in anxiety-like behaviour and memory impairment in adulthood, demonstrating that PhB effects are conserved among rodents^19^. Taking these behavioural and cellular outcomes together it shows that PhB given to treat neonatal seizures does not mitigate the negative neurological outcomes in later life, but in fact acts as a pheno-mimetic similar to hypoxia itself.

It has been shown that there are long-term increases in brain hyper-excitability following seizures in the neonatal period and thus a risk factor for epilepsy later in life^34,35^. Indeed, 18% of neonates with seizures will develop epilepsy in the first year of life^36^. Our study found that early-life exposure to hypoxia exacerbates responses to the chemoconvulsant KA in later life and PhB does not mitigate the hypoxia response to KA. The effect of neonatal seizures on neuronal excitability was consistent with other findings, including our own^21^, with a marked decreased threshold for further seizures when challenged later in life. Also, we observed that exposure of pups to PhB alone was sufficient to increase seizure vulnerability weeks later, consistent with previous results in PhB treated P3–21 neonatal rats that later received the convulsant pentylenetetrazol (PTZ) at 6–7 weeks old^37^. How does PhB induce this hyper-excitable phenotype later in life? PhB exposure has been shown to damage GABAergic neurons and disrupt neurogenesis in the neonatal period in rodents^12^. This reduction in the inhibitory GABAergic neurons may explain the formation of pro-excitatory circuits.

Surprisingly, when neuronal death was analysed after KA treatment in adulthood, exposure to PhB at P7 had an apparent beneficial effect compare to the hypoxia insult. This is a surprising finding; however recently, PhB has been reported to be protective in the CA3 subfield of the hippocampus after pilocarpine induced status epilepticus in rats, and no effect was observed in the CA1 and DG^30^. This beneficial effect of PhB may be due to micro-anatomical changes. Ultrastructural changes in neuronal and synaptic structure may exist in addition to lasting shifts in gene expression which alter excitability. These, rather than overt damage, would be sufficient to account for behavioural phenotypes and electrographic hyper-excitability.

In the present study, we aimed to analyse the effects of PhB as a treatment of hypoxia-induced seizures. Even though we did not see an improvement in acute damage and neurological outcomes, we cannot exclude that PhB may have a beneficial effect as an adjuvant of therapeutic hypothermia (TH). In fact, in a recent publication, Krishna and colleagues have shown that PhB has a neuroprotective effect when used as an adjuvant TH in a rat model of hypoxia-ischemia encephalopathy^38^.

The current study presents some limitations. First, our study did not control for maternal care and also our inability to distinguish responders and non-responders to PhB in the long-term studies. Parental behaviour has been shown to influence the neurological development of offspring^39^. We observed that hypoxia and PhB- treated P7 pups were slower to gain weight after procedures, perhaps reflecting poor maternal care. Efforts were to limit this, all pups were away from dams for equal time (no longer than 30–60 min), minimal handling and prompt return to the home cage. While we cannot exclude a contribution of poor maternal care in lasting neurodevelopmental outcome, previous work has shown that transient poor maternal care does not affect the neurodevelopment of offspring^39^. Another limitation of the present study is our inability to differentiate between responders and non-responders to PhB in the long-term study. This is difficult to control for since phenobarbital causes motor sedation and electrographic uncoupling that would make it difficult to differentiate between responders and non-responders by behavioural scoring of pups at the time of hypoxia-induced seizures in the present study. We have not seen differences between individuals treated with PhB; however, we cannot discard differences between responders and non-responders. Further analysis will be necessary to evaluate long-lasting benefits of PhB in responders compared to non-responders, and determine the sub-group of infants who will greatly benefit from PhB treatment.

Finally, our results highlight the need for rigorous testing of novel anticonvulsant agents in infants. Although some drugs may halt seizures in neonates, due to the critical developmental period in which they are given, they may produce unforeseen long lasting outcomes, far out-lasting their initial administration. This is further highlighted with recent clinical trials testing bumetanide as a possible anti-seizure drug in neonates^40^.

In conclusion, the present study comprises a comprehensive characterization of PhB treatment effects after hypoxia in an age-appropriate model of neonatal seizures in mice. We found that a single dose of PhB has the same long-lasting effects as hypoxia-seizures, and PhB does not mitigate the long-lasting effect of seizures during the neonatal period. However, we cannot assume that PhB has not a detrimental effect of hypoxia, because we may see a floor effect in some our experimental settings (e.g. novel-object location). Further, combining our etiologically and clinically relevant animal model of hypoxia induced neonatal seizures and its use of the mouse strain C57BL/6J, it allows us to fully interrogate the effects of new agents on acute neonatal seizures, its long term effects and also the molecular mechanisms behind these effects.

## Materials and Methods

### Mouse model of neonatal hypoxia-induced seizures and phenobarbital treatment

Animal experiments were performed in accordance with the principles of European Communities Council Directive (86/609/EEC, 2010/63/EU), under license (REC#1132b) from the Department of Health and Health Products Regulatory Authority (Ireland) and procedures were approved by the Research Ethics Committee of the Royal College of Surgeons in Ireland. Neonatal litters of C57BL/6J mice (weight, 4–6 g; age, postnatal day 6.5–7.5 (P7)), were obtained from the Biomedical Research Facility, RCSI. Pups were kept with their dams in a barrier-controlled facility on a 12 h light-dark (7am–7pm) standard cycle with access to food and water ad libitum. All experiments were performed during the light cycle. To induce global hypoxia, male and female pups were randomly placed in a hypoxic chamber and exposed to a premixed gas containing 5% O_2_/95% N_2_ for 15 min at 34 °C. Normoxic control pups were placed in the chamber at 21% oxygen (room-air) for the same period of time (Fig. 1A).

Immediately following 15 min of normoxia or hypoxia, pups received one single intraperitoneal (i.p.) dose of vehicle (100 μL 0.9% saline) or phenobarbital (100 μL PhB, 25 mg.kg^−1^, Martindale Pharmaceuticals, U.K.). For electrographic analysis, pups were prepared for EEG recordings (as described below), and recorded during hypoxia and 20–30 min post-hypoxia. Pups that under-went acute EEG analysis were euthanized immediately after recordings. For histology and analysis of the long-term effects of PhB, pups were subjected to hypoxia without EEG electrodes and returned to dams (Fig. 1A).

In summary, four experimental groups were generated: normoxic pups receiving vehicle or PhB (control and PhB groups); and hypoxic pups receiving vehicle or PhB (hyp and hyp-PhB groups). For each experimental paradigm, pups from each litter were blindly randomized between the four groups. Numbers of pups and percentage of males in each experiment are described in Table 1.

**Table 1:** Number of mice and percentage of males have been annotated per every individual parameter and group.

	Control		PhB		Hypoxia		Hypoxia-PhB	
N Number/%males								
Figure 1D,E,F	6	66.0	6	50.0	8	62.5	8	50.0
Figure 1G	7	42.8	7	42.8	7	57.0	8	50.0
Figure 2B	7	42.8	7	42.8	7	57.0	8	50.0
Figure 3B,C,E,G	11	63.7	13	53.8	12	58.3	11	61.5
Figure 4C,D,E,F	10	50.0	12	50.0	9	55.5	9	55.5
Figure 5B,C	7	57.0	7	43.0	6	50.0	6	50.0

Left side of the table shows the specific figure and subsection. Control, hypoxia and hypoxia-PhB groups are annotated from left to right. In every square, the left column shows the “n number” in the particular experiment and condition, and the right side the percentage of males in the particular experiment and condition. For details on the particular experiment, refer to manuscript and figure legends.

### Electroencephalography recordings in P7 pups

Mice were placed in a stereotaxic frame and anesthetized with isoflurane/oxygen (5% for induction, 2–3% maintenance). Temperature was maintained with a heat-pad (Harvard Apparatus Ltd., U.K.). Isoflurane exposure was limited to 8–10 min, where three partial craniectomies were performed and electrodes (E/363/20, Bilaney Ltd., U.K.) secured into the skull with dental cement. One electrode was placed in each temporal cortex (−*10* *mm AP and* +/−*2*.*5* *mm* *ML from Bregma)* and one reference in the cerebellum. EEG was recorded using a Grass Comet XL digital EEG-amplifier and digitalized with Twin software using Notch filter (Grass Technologies Ltd., Warwick, RI). In P7 pups, seizures were induced by hypoxia as previously described^21^.

For analysis of EEG data, filtered files were uploaded to LabChart Pro (V7, ADInstruments Ltd.). Seizures were defined as electrographic polyspike discharges ≥5 Hz, ≥2x baseline EEG amplitude and lasting ≥5 s. EEG total power ((μV^2^) is a function of EEG amplitude over time) was analysed by integrating frequency bands from 0 to 100 Hz. For these analyses, post-hypoxia traces were selected and the values were normalised to the baseline of each animal (pre-hypoxia). Artefact or EEG noise were identified and excluded from analysis. The number and duration of seizures (measured as the time from first spike to last spike) were calculated per hypoxic and post-hypoxic episode of EEG recording. Total seizure burden was calculated as the accumulative time that pups or mice were having electrographic polyspikes discharges. Power spectral density heat maps were generated within LabChart (spectral view), with the frequency domain filtered from 0 to 80 Hz and the amplitude domain filtered from 0 to 40 mV. For the classification of PhB-responders and non-responders, EEG was evaluated post-phenobarbital injection (range 0–100 Hz). Pups with a reduction of electrographic seizures were considered to responders to Phenobarbital.

Behavioural seizures were scored using the Morrison scale (1996)^21^: Score 0: Normal behaviour; Score 1: Immobility and myoclonic jerks; Score 2: Rigid posture; Score 3: Circling, repetitive pedalling movements, head bobbing and tail extension; Score 4: spasms, forelimb clonic-tonic seizures, loss of posture with hyperventilation; Score 5: Repeated stage 4.

### Electroencephalography recordings in 6 weeks old mice

To test for long-term alterations in seizure susceptibility, additional mice were challenged with systemic kainic acid (KA; 15 mg.kg^-1^, intraperitoneal; i.p.) five weeks after experiments at P7. Mice were placed in a stereotaxic frame and anesthetized with isoflurane/oxygen (5% for induction, 2–3% maintenance). Temperature was maintained with a heat-pad (Harvard Apparatus Ltd., U.K.). Three partial craniectomies were performed and electrodes (E/363/20, Bilaney Ltd., U.K.) secured into the skull with dental cement. One electrode was placed in each temporal cortex (19.*5 mm and* +/−*3*.*5* *mm* *ML from Bregma)* and one reference in the cerebellum. EEG was recorded using a Grass Comet XL digital EEG-amplifier and digitalized with Twin software using Notch filter (Grass Technologies Ltd., Warwick, RI). Analysis of EEG was performed as describe above^21^. Behavioural scores were based on a 6-point Racine-like scale, as described previously^41^. Score 0, normal activity; Score 1, immobility or rigid posture; Score 2, stiffened and tail extension; Score 3, forelimb clonus or head bobbing; Score 4, rearing; Score 5, rearing and falling; and Score 6, tonic-clonic seizures with loss of posture or jumping. All analyses were carried out by observers blind to the experimental condition.

### Cognitive and behavioural testing

Cognitive and behavioural testing was performed four weeks after experiments at P7 (P35 mice). Locomotor activity was examined using the open-field assay (30 × 30 × 20 cm) prior to the novel object-location task (see below) for 10 min. Total distance travelled, velocity, freezing episodes, number of crossings and average time spent in specific central/peripheral zones of the arena were quantitatively analysed using video tracking (Ethovision, Tracksys, Nottingham, U.K.).

Anxiety-related behaviour was assessed using the light/dark box^20^. The test apparatus consisted of an open glass box (30 × 30 × 20 cm) connected to an acrylic dark box (30 × 15 × 20 cm) via an entrance. The light area was illuminated from above by a white lamp bulb (60 W/600 lux). Mice were placed in the centre of the light area, facing away from the entrance of the dark area, and were allowed to explore the apparatus for 10 min. Anxiety-related behaviour was quantitatively assessed in terms of latency to enter the dark area by Ethovision software.

Hippocampal memory function was assessed using a novel object-location test as described previously^26,28^. The test was carried out over two consecutive days in 10 min sessions. Day 1 (habituation day), mice explored the arena without objects (10 min) followed by 3 sessions with two objects; on day 2 (test day), mice were placed in the arena with one of the objects moved to a novel position (Fig. 2F). Object exploration was manually recorded and defined as the time mice were interacting with the objects with their nose or paw within 1 cm^21^. To calculate the percentage of time with the novel-object, the following equation was used: (A)/(A + B)^*^100. A = Time spend with novel object. A + B = Time spend with both objects. Cognitive and behavioural testing was analysed by an observer blinded to the treatment.

### Histological Analyses

For histological analyses 72 h post hypoxia (at this time point we have previously observed the maximum levels of reversible neuronal damage^21^), pups were transcardially perfused with PBS followed by 4% PFA/2.5% glutaraldehyde in 0.1 M phosphate buffer, and post-fixed in the same solution. Brains were then sectioned using a Vibratome at 50 μm (Leica VT1000), kept in a cryo-protected solution and mounted onto glass slides.

Silver Staining was performed to evaluate potential reversible neuronal injury. Briefly, free-floating sections were processed using a method developed by Gallyas *et al*.^24,42^, with minor modifications. Sections were dehydrated and esterified with 100% 1-propanol containing 0.8% sulfuric acid. Upon rehydration and 1% acetic acid treatment, sections were developed in 0.2% AgNO_3_, 0.25% NH_4_NO_3_, 2% tungstosilicic acid and 0.4% formaldehyde. Sections were dehydrated, cleared and covered with DPX (Sigma-Aldrich). Semi-quantitative analysis of Silver Staining was performed under 20X lens magnification of the hippocampus. The hilus from two consecutive slides were counted per condition and the average number of positive cells from both slides was calculated^21^.

Fluoro-Jade B staining was performed to evaluate permanent neuronal injury after kainic acid seizures. Mice were sacrificed and perfused with PBS followed by 4% paraformaldehyde (PFA). Brains were collected and post-fixed in 4% PFA overnight, embedded in 4% agarose and cut at 50 μm. Coronal sections were mounted onto glass slides and dried at 37 °C overnight. Neuronal injury was analysed using Fluoro-Jade B (FJB) staining as previously described^27^. Semi-quantitative analysis FJB staining was carried out under an epi-fluorescence microscope at 20X lens magnification (Nikon 2000s). Images were split into individual colour component (red, green and blue) using Image J program, this image was then inverted to produce highlighted image shown (Fig. 5A)^25^. Individual cells were counted from two consecutives slides within hippocampal sub-field using standard subfield boundaries^33^ by an observer blinded to the treatment.

### Data analysis

All data are presented as Box and Whiskers plots to show first and third quartiles, median and minimum and maximum of all the data. All statistical tests were carried out in the R statistical environment (https://cran.r-project.org). Group comparisons were made using the non-parametric Kruskal-Wallis rank sum test to determine if differences between groups occurred, as the datasets were not normally distributed. Where differences were found, a Wilcoxon rank sum test was used to perform pairwise comparisons between the control and the three conditions (PhB, Hyp, Hyp-PhB) to determine that each group is performing at a significantly different rate than would be expected by chance, and the p-values were adjusted for multiple testing using the Bonferroni correction. For Fig. 1E,F, a Wilcoxon rank test was carried to valorate differences between the hypoxia and hypoxia-PhB groups. The appropriate p-values and W-values are incorporated into Table 2 and p-value of <0.05 was considered significant.

**Table 2:** Statistical information for every individual experiment and parameter.

	Wilcoxon test (W)	Kruskal-Wallis
Figure 1D		5.76
Figure 1E	47	Not applicable
Figure 1F	56.2	Not applicable
Figure 1G	W_PhB_ = 87; W_Hyp_ = 90.5; W_Hyp-PhB_ = 89.5	19.29
Figure 2C	W_PhB_ = 2.5; W_Hyp_ = 3.4; W_Hyp-PhB_ = 3.1	4.95
Figure 3B	No difference	1.72
Figure 3C	No difference	3.72
Figure 3E	W_PhB_ = 84; W_Hyp_ = 124; W_Hyp-PhB_ = 107	10.16
Figure 3G	W_PhB_ = 115; W_Hyp_ = 114; W_Hyp-PhB_ = 124	15.84
Figure 4D	W_PhB_ = 4; W_Hyp_ = 0; W_Hyp-PhB_ = 0	18.20
Figure 4F	W_PhB_ = 24; W_Hyp_ = 15; W_Hyp-PhB_ = 7	8.29
Figure 5B	W_PhB_ = 33.4; W_Hyp_ = 34.8; W_Hyp-PhB_ = 34.8	1.96
Figure 5C (CA3,top)	No difference	1.67
Figure 5C (Hilus, below)	No difference	1.20

## Electronic supplementary material

Supplementary Figure 1 and 2
