# Supplementary material for: Complex spectrum of phenobarbital effects in a mouse model of neonatal hypoxia-induced seizures
Source: Sci Rep. 2018 Jul 3;8:9986. doi: 10.1038/s41598-018-28044-2 (PMC6030182; doi:10.1038/s41598-018-28044-2)
Supplement: Supplementary file 1 — Supplementary Figure 1 and 2 [file 41598_2018_28044_MOESM1_ESM.docx]

**Complex Spectrum of phenobarbital effects in a mouse model of neonatal hypoxia-induced seizures**

*New suggested title: Behavioral, electrographic and histopathological effects of phenobarbital in a mouse model of neonatal hypoxia-induced seizures*

Sean MM Quinlan, Natalia Rodriguez-Alvarez, Eleanor J Molloy, Stephen F Madden, Geraldine B Boylan, David C Henshall and Eva M Jimenez-Mateos.

(Supplementary information (Supplementary Figures 1-2))

**Supplementary Figure 1.** Analysis of seizure burden during hypoxia and post hypoxia period in the Hyp and Hyp-PhB groups of each individual P7 pup. Duration of seizure burden during the 15 min of hypoxia and post-hypoxia period in the hypoxia group (A) and hypoxia-PhB group (B). Note: Dots link by a line represents an individual P7 pup. Green line represents the four P7 pups were considered to respond to phenobarbital. (C) Ratio of the seizure burden between the post-hypoxia period and hypoxia period. *p<0.05, compared to hypoxia and no-responders to Phenobarbital.

**Supplementary Figure 2.** Analysis of the first five minutes of the

Open Field to evaluate anxiety-like behavior. Distance travel (A), time spent in the periphery (B), velocity (C), freezing time (D) and number of crossing between the center and periphery area (E) was evaluated as an index of anxiety-like behavior. Note: No differences were observed between experimental groups.
